# Muscle Activity Characteristics of the Pronator Teres during Throwing in Baseball Pitchers: A Pilot Study

**DOI:** 10.3390/healthcare11040618

**Published:** 2023-02-19

**Authors:** Akihiro Tamura, Masami Saito

**Affiliations:** Department of Physical Therapy, School of Health Sciences at Narita, International University of Health and Welfare, Narita 286-8686, Japan

**Keywords:** electromyography, elbow joint, forearm, injury prevention

## Abstract

The pronator teres muscle is a major dynamic stabilizer of elbow valgus stress during throwing. This study aims to investigate pronator teres muscle activation during breaking ball pitching in baseball pitchers. Twelve male college baseball players with more than eight years of baseball experience were included in this study. A wireless surface electromyography (EMG) system was used to measure the activation of the forearm muscles and record EMG data during fastball and curveball pitching. Peak pronator teres muscle activation during curveball pitching was greater than that during fastball pitching (*p* = 0.03). There was no difference in the muscle activation of the other forearm muscles (*p* > 0.05). These results indicate that increased muscle activity in the pronator teres may contribute to stiffness and induce pronator teres syndrome or medial elbow injuries related to the overuse of the pronator teres, especially during curveball pitching. Controlling curveball throws contributes to player coaching and conditioning for the prevention of elbow joint disorders and pronator teres syndrome.

## 1. Introduction

Baseball is a sport that is played by a large number of players, not only in Japan but also worldwide. At the same time, sport injuries frequently occur during baseball games, and injury prevention methods are widely discussed. In particular, it is known that the motion characteristics during baseball differ greatly due to the positional differences between pitchers and batters [1,2]. Therefore, in order to prevent injuries in baseball, it is very important to fully consider the player’s position and role in the team.

Most sports injuries associated with the throwing movement in baseball are likely to occur in the shoulder and elbow joints, which are reportedly caused by extremely high mechanical stress on these joints [3,4]. High injury rates (26–35% per a year) of young baseball players in elementary, junior high, and high school, who are in the developmental stage of the musculoskeletal system, lead to shoulder and elbow joint disorders due to accumulated mechanical stress associated with the pitching motion [5]. Baseball players in the growth stage often suffer from osteochondritis dissecans of the capitellum on the lateral side and medial epicondyle avulsion fractures [6,7,8]. In addition, disorders of the muscles, tendons, ligaments, and nerves, such as the elbow joint flexors, iliopsoas tendon, brachioradialis muscle, and medial and lateral collateral ligaments, increase in adolescent high-school baseball players [9]. Therefore, it is important to elucidate the factors that lead to the onset of injuries in pitchers of various ages, as well as examine and investigate preventive strategies.

Baseball pitchers need to pitch various types of breaking balls, including curved balls, to strategically prevent the ball from being hit; however, throwing a significant number of breaking balls may increase the risk of elbow joint disorders. Previous studies have indicated that forearm muscle activation and mechanical force applied to the elbow change by applying a strong spin to the ball when pitching straight or breaking balls [10,11]. Research focusing on curveballs has revealed that a spin on the ball is applied by the thumb, middle finger, and index finger, which is significantly different from the finger kinematics used when throwing a fastball [12]. Furthermore, the motion of the forearm and fingers when throwing a curveball revealed that the first tends to assume a supine position at ball release because of the spin added to the ball by the fingers [13,14]. In addition, it has been reported that increasing the forearm supination moment is related to an increase in the elbow varus moment during curveball pitching [14]. Therefore, curveball pitching may cause excessive contractions of the forearm flexor muscles associated with an increase in elbow varus moment. As a result, this could lead to adding excessive stress to the medial part of the elbow joint.

Among the forearm flexor muscles, the pronator teres muscle is one of the major dynamic stabilizers of elbow valgus stress during throwing movements [15,16]. Recent research has clarified that osteochondritis dissecans and medial elbow injuries are related to the stiffness of the pronator teres muscle in the throwing arm of baseball pitchers [17]. It has been suggested that pronator teres syndrome, recognized as a representative proximal forearm median neuropathy [18], is caused by compression of the median nerve by the pronator teres muscle in the forearm [16,19]. This syndrome occurs by producing excessive contractions of the forearm flexor muscles with large mechanical stresses at the medial elbow joint during throwing movements [20]. Therefore, overuse of the pronator teres muscle may cause serious throwing disorders in the elbow joint of baseball pitchers by causing medial elbow injuries or median neuropathy.

Previous studies have reported the forearm, wrist, and finger motion characteristics of various throwing types [12] and differences in pelvis, torso, and upper extremity muscle activation between different breaking balls in baseball pitchers [21]. In spite of this, it has not been clarified whether the pitching of a breaking ball causes excessive muscle contractions of the forearm flexors. Moreover, no studies have investigated the effects of breaking ball pitching on the pronator teres muscle or considered the effects of curveball pitching on medial elbow injuries, including pronator teres syndrome.

This study aims to investigate pronator teres muscle activation during breaking ball pitching in baseball pitchers as a pilot study to assess the risk of pitching injury on a larger scale. We hypothesize that the muscle activation of the pronator teres muscle in curveball pitching was greater than that in fastball pitching. Our study could contribute to player coaching and conditioning focused on the prevention of elbow joint disorders and pronator teres syndrome.

## 2. Materials and Methods

### 2.1. Study Design

This pilot cross-sectional study was conducted between August and December 2020 at a gymnasium of the International University of Health and Welfare, Narita Campus, Chiba, Japan. Participants were recruited between August 2020 and October 2020 at the International University of Health and Welfare, Narita Campus, Chiba, Japan. The study protocol was approved by the International University of Health and Welfare Ethics Committee (Approval No. 19-Io-104) and was conducted in accordance with the tenets of the Declaration of Helsinki. All participants provided written informed consent prior to testing.

### 2.2. Participants

Twelve male college baseball players with more than eight years of baseball experience were included in this study (height: 171.7 ± 4.8 cm; weight: 64.6 ± 4.5 kg; age: 20.5 ± 1.5 years; baseball experience: 9.9 ± 1.2 years). Before inclusion in this study, all participants were required to answer a questionnaire about their physical characteristics (age, height, and weight), current medical information, and medical history. The inclusion criteria were pitchers in main or sub positions while playing baseball, age ≥ 18 years, baseball experience of >8 years including high-school baseball, and absence of current pain in the upper extremities or trunk during ball throwing. The exclusion criteria were any history of orthopedic surgery in the upper extremities.

### 2.3. Instrumentation and Measurement Protocols

**Figure 1:**
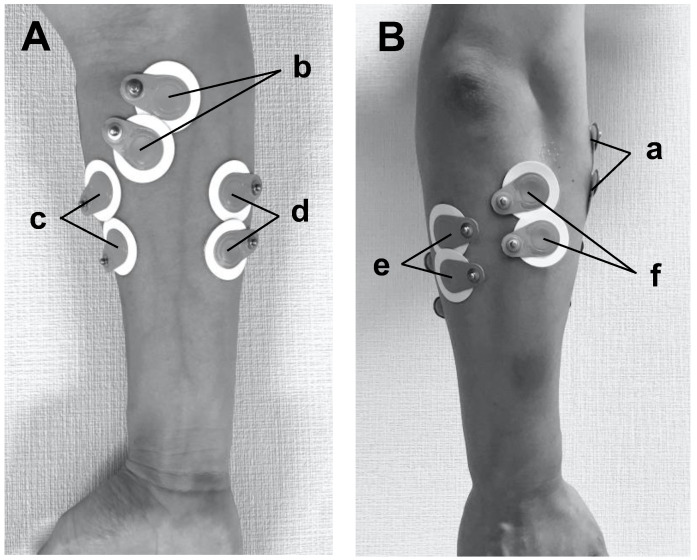
Surface electrodes placement. (**A**,**B**) represent the dorsal (posterior) and palmar (anterior) views of the right forearm, respectively: (a) brachioradialis muscle, (b) pronator teres muscle, (c) flexor carpi radialis, (d) flexor carpi ulnaris, (e) extensor carpi ulnaris, (f) extensor carpi radialis.

A wireless surface electromyography (EMG) system (TeleMyo DTS, Noraxon Co., Arizona, USA) was used to measure muscle activation of the forearm during the pitching motion. The sampling frequency was set to 1500 Hz, and the bandpass filter was set to 10–500 Hz. Surface electrodes (Blue Sencer SP-00-S, Ambu, Denmark) were placed on the bellies of six muscles, namely the brachioradialis muscle (Figure 1B-a), the pronator teres muscle (Figure 1A-b), the flexor carpi radialis (Figure 1A-c), the flexor carpi ulnaris (Figure 1A-d), the extensor carpi ulnaris (Figure 1B-e), and the extensor carpi radialis (Figure 1B-f), at a 2 cm distance of between the electrodes. TeleMyo DTS wireless sensors (Noraxon Co., Arizona, USA) were connected to the respective surface electrodes, then they were attached to the designated positions with double-sided tape and secured in place with flexible athletic tape.

Maximum voluntary contractions (MVCs) were measured in accordance with the functional characteristics of the brachioradialis muscle, pronator teres muscle, flexor carpi radialis, flexor carpi ulnaris, extensor carpi ulnaris, and extensor carpi radialis. Participants slowly increased the force to maximum effort for 3 s and then relaxed again for 3 s. The MVC trials were repeated thrice per muscle.

After a standard warm-up, which included comfortable pitching in a gymnasium, all participants were required to perform a pitching motion toward a catcher positioned 18.44 m away from the pitcher’s plate. All the subjects were assigned in a random order for throwing fastballs or curveballs. They pitched five fastballs and five curveballs to the best of their abilities. The recovery intervals were set to a minimum of 30 min between the fastball and curveball pitching trials.

### 2.4. Phases of the Pitching Cycle

Pitching motion was defined as six phases according to previous studies as follows: wind up; early cocking; late cocking; acceleration; deceleration; and follow through [22,23]. The wind-up phase was defined as the phase that begins with the pitcher’s first movement from the static position of facing the batter with both feet on the mound and is completed when the lead leg reaches maximum knee height. The early cocking and late cocking phases were defined as the phases that begins with the front foot striking the ground and ends with maximum shoulder external rotation at 150 to 180°. The acceleration and deceleration phases were defined as the phases that take place from maximal external rotation to the moment of ball release and between the time of ball release and maximal humeral-head internal rotation with elbow extension, respectively. The follow-through phase was defined as the phase where the body continues to move forward until the arm has ceased motion.

### 2.5. Data Analysis

The muscle activations (μV) of the brachioradialis muscle, pronator teres muscle, flexor carpi radialis, flexor carpi ulnaris, extensor carpi ulnaris, and extensor carpi radialis during fastball and curveball pitching motions were recorded from the maximum external rotation position in the end of cocking phase to the end of the follow-through phase. The root mean square (RMS) of the EMG calculated from the data was obtained and normalized using the MVC values for the muscles; thus, the EMG measurement units were expressed relative to the MVC (%MVC). All data were averaged and the peak values across each participant’s fastball and curveball trials were calculated. The EMG variables were converted to a percentage from the maximum external rotation position in the late cocking phase to the end of the follow-through phase, with 0% representing maximum shoulder external rotation and 100% representing the end of the follow-through phase.

### 2.6. Statistical Analysis

The Kolmogorov–Smirnov test was used to confirm that the EMG data were normally distributed (*p* > 0.05). Depending on whether the physical characteristics and EMG data were normally distributed, paired t-tests and Wilcoxon signed-rank tests were used to identify differences in the variables between fastball and curveball pitching in each participant. A probability *p* value of <0.05 was considered statistically significant. Cohen’s d effect sizes (ESs) were calculated for all analyses and showed the magnitude of the differences between fastball and curveball pitching. Data analyses were conducted using IBM SPSS Statistics for Windows version 24.0. (IBM Corporation, Armonk, NY, USA). One-dimensional statistical parametric mapping (SPM[t]) paired t-tests were performed to compare each individual point of the averaged EMG curves from the late cocking phase to the follow-through phase between fastball and curveball pitching. The threshold of significance was set at α = 0.05 for all analyses. SPM(t) paired t-tests were conducted using the open-source SPM1d code (www.spm1d.org, accessed on 6 January 2023) in MATLAB_R2020b (MathWorks Inc., Boston, MA, USA).

## 3. Results

### 3.1. Average EMG Values

**Figure 2:**
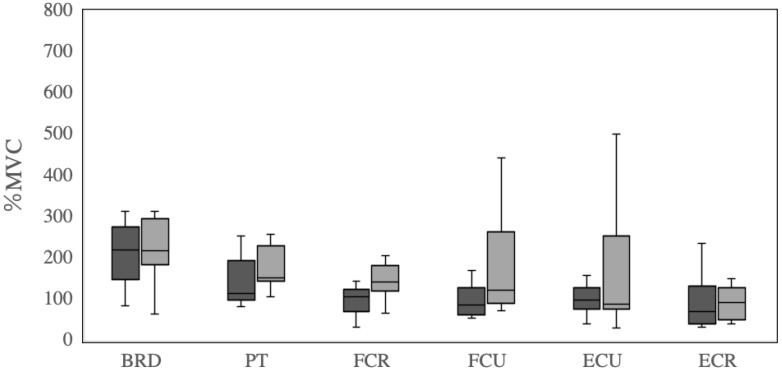
Comparison of EMG average values (%MVC) in forearm muscles during fastball and curveball pitching Horizontal black lines represent median values within each box. Boxes extend from the lower quartile to the upper quartile of the distribution of values during fastball and curveball pitching, respectively. The whiskers indicate the minimum value to the lower quartile and then the upper quartile to the maximum value. EMG data (%MVC) are presented on the *y*-axis. BRD: brachioradialis muscle; PT: pronator teres muscle; FCR: flexor carpi radialis; FCU: flexor carpi ulnaris; ECU: extensor carpi ulnaris; ECR: extensor carpi radialis.

The average EMG data of brachioradialis muscle, pronator teres muscle, flexor carpi radialis, flexor carpi ulnaris, extensor carpi ulnaris, and extensor carpi radialis during fastball and curveball pitching are shown in Table 1 and Figure 2. There was no difference in the peak variables in brachioradialis muscle (234.8 ± 125.8 and 220.8 ± 74.5 %MVC, *p* = 0.71, ES = 0.12), pronator teres muscle (165.4 ± 114.4 and 192.7 ± 91.9 %MVC, *p* = 0.20, ES = −0.42), flexor carpi radialis (114.9 ± 76.6 and148.1 ± 42.5 %MVC, *p* = 0.14, ES = −0.49), flexor carpi ulnaris (114.3 ± 82.9 and 181.0 ± 134.7 %MVC, *p* = 0.09, ES = −0.57), extensor carpi ulnaris (135.7 ± 127.1 and 155.9 ± 144.0 %MVC, *p* = 0.77, ES = −0.09), and extensor carpi radialis activations (104.9 ± 90.5 and 111.9 ± 80.1 %MVC, *p* = 0.87, ES = −0.05) between fastball and curveball pitching.

### 3.2. Peak EMG Values

**Figure 3:**
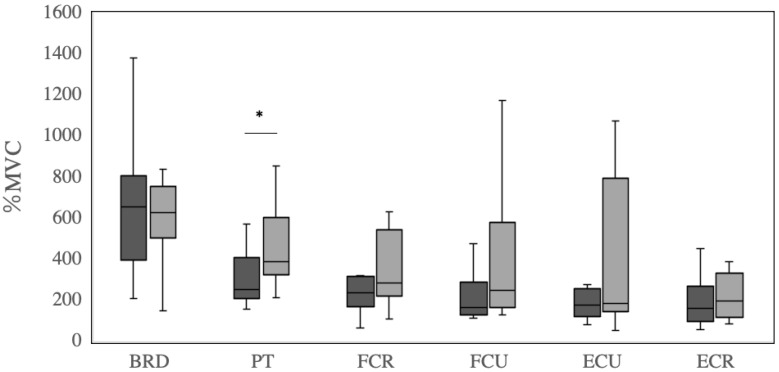
Comparison of EMG peak values (%MVC) in forearm muscles during fastball and curveball pitching. Horizontal black lines represent median values within each box. Boxes extend from the lower quartile to the upper quartile of the distribution of values during fastball and curveball pitching, respectively. The whiskers indicate the minimum value to the lower quartile and then the upper quartile to the maximum value. EMG data (%MVC) are presented on the *y*-axis. *: statistical significance (*p* < 0.05); BRD: brachioradialis muscle; PT: pronator teres muscle; FCR: flexor carpi radialis; FCU: flexor carpi ulnaris; ECU: extensor carpi ulnaris; ECR: extensor carpi radialis.

The peak EMG data of the brachioradialis muscle, pronator teres muscle, flexor carpi radialis, flexor carpi ulnaris, extensor carpi ulnaris, and extensor carpi radialis during fastball and curveball pitching are shown in Table 2 and Figure 3. Peak pronator teres muscle activation during curveball pitching (450.4 ± 193.7 %MVC) was greater than that during fastball pitching (340.1 ± 226.2 %MVC, *p* = 0.03, ES = −0.79). There were no difference in the average brachioradialis muscle (658.4 ± 329.3 and 1598.1 ± 218.3 %MVC, *p* = 0.60, ES = 0.17), flexor carpi radialis (260.2 ± 147.3 and 346.0 ± 175.8 %MVC, *p* = 0.18, ES = −0.46), flexor carpi ulnaris (268.6 ± 260.5 and 428.6 ± 429.2 %MVC, *p* = 0.20, ES = −0.47), extensor carpi ulnaris (308.6 ± 438.8 and 380.8 ± 371.8 %MVC, *p* = 0.73, ES = −0.11), and extensor carpi radialis activations (233.2 ± 229.1 and 269.3 ± 233.1 %MVC, *p* = 0.76, ES = 0.10) between fastball and curveball pitching. However, in the flexor carpi ulnaris and extensor carpi ulnaris in curveball pitching, the wide distribution over the interquartile range and to the maximum indicated that there was a variation in the distribution of mean values.

### 3.3. Time-Series EMG Values

**Figure 4:**
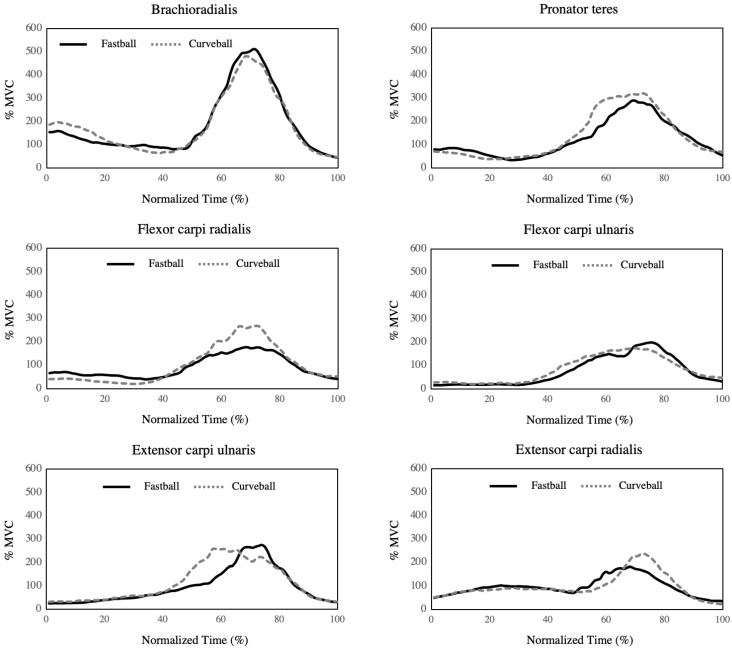
Time-series data of muscle activations during fastball and curveball pitching. Mean values of EMG data (%MVC) during fastball (black lines) and curveball (dotted gray lines). The normalized time (%) was defined as duration from the late cocking phase to the follow-through phase, with 0% representing the maximum shoulder external rotation and 100% representing the end of the follow-through phase and is presented on the *x*-axis. EMG data (%MVC) are presented on the *y*-axis.

The time-series EMG data of brachioradialis muscle, pronator teres muscle, flexor carpi radialis, flexor carpi ulnaris, extensor carpi ulnaris, and extensor carpi radialis during fastball and curveball pitching are shown in Figure 4. No between-group differences in these variables were observed at any time point during in all muscles between fastball and curveball pitching (*p* > 0.05).

## 4. Discussion

Baseball pitchers need to pitch various types of breaking balls, including curved balls, to strategically prevent the ball from being hit. Throwing many breaking balls, including curveballs, may increase the risk of elbow joint disorders and cause excessive contractions of the forearm flexor muscles associated with increasing elbow varus moment. This study aimed to investigate the pronator teres muscle. Such excessive contractions have been activated using a surface EMG system during breaking ball pitching in baseball pitchers as a pilot study to assess the risk of pitching injury on a larger scale. The research hypothesis was that muscle activation of the pronator teres in curveball pitching was greater compared to that in fastball pitching.

Measurements of muscle activation by a surface EMG system are useful for evaluating the characteristics of specific motions in various sports [24,25]. Regarding baseball pitching, muscle activities in the upper extremity during throwing motions have been investigated for various purposes and used to prevent injury and improve athletic performance [26,27]. Therefore, in this study, a surface EMG system was used to evaluate pronator teres muscle activation during breaking ball pitching in baseball pitchers to assess the risk of pitching injury.

The results of this study reveal that average muscle activation of the pronator teres muscle in curveball pitching was greater than that in fastball pitching. In a previous study, analyses of the motion of the forearm while throwing a curveball revealed that the forearm tended to assume a supinate position during ball release [13,14]. This characteristic motion is related to an increase in elbow varus moment in curveball pitching [14]. In addition, the pronator teres muscle extends between the radius and medial epicondyle of the humerus and plays the role of the active stabilizer of the elbow during valgus stress with a function of radioulnar joint pronation [28,29]. Therefore, during curveball pitching, it is considered that the pronator teres muscle acts to control valgus stress in the elbow as the valgus moment of the elbow increases. In addition, the throwing arms of players with capitellar osteochondritis dissecans and medial elbow injuries show stiffness in the pronator teres muscle [17]. This study suggests that the stiffness of the pronator teres muscle may be the result of increased muscle activity during pitching motion.

The pronator teres muscle is one of the major dynamic stabilizers of elbow valgus stress during throwing [15,16]. Dididze et al. (2022) clarified that osteochondritis dissecans and medial elbow injuries are related to the stiffness of the pronator teres muscle in the throwing arm of baseball pitchers [17]. In addition, Loomer (1982) reported on the development of forearm muscle syndrome in athletes, which is recognized as a representative proximal forearm median neuropathy [18]. Pronator teres syndrome is caused by the compression of the median nerve by the pronator teres muscle with excessive contractions of the forearm flexor muscles during throwing sports [16,19,20]. Therefore, greater muscle activation of the pronator teres muscle during pitching may cause serious throwing disorders in the elbow joint of baseball pitchers by causing medial elbow injuries or median neuropathy. The results of the present study suggest that increased muscle activity in the pronator teres during curveball pitching may contribute to the stiffness of the pronator teres muscle and, consequently, to pronator teres syndrome. These results indicate that it is important to recognize that increased muscle activity in the pronator teres may induce pronator teres syndrome or medial elbow injuries related to the overuse of the pronator teres muscle as a result of increased muscle activity in the ileus, especially during curveball pitching.

Forearm muscle activation, excluding the pronator teres muscle, did not differ between fastball and curveballs in this study. In previous kinematic studies, the forearm of the throwing arm was in a supine position during the ball release phase of curveball pitching [11,12]. Therefore, it is considered that the pronator muscle is forced to contract excessively in the supinated position in that phase, while the brachioradialis muscle, flexor carpi radialis, flexor carpi ulnaris, extensor carpi ulnaris, and extensor carpi radialis are less affected by forearm supination due to the structural and functional characteristics of these muscles. In general, contractions in lengthened muscles are known to result in greater force produced by the muscle according to the force–length relationship [30]. Additionally, lengthening contractions result in injury to skeletal muscle fibers [31]. This suggests that the forearm supine position, which is an extended position of the pronator teres muscle, during curveball pitching, which involves an extended position of the pronator teres muscle, caused excessive muscle activation of the pronator teres at ball release. Therefore, in the present study, an increase in muscle activation was observed specifically in the pronator teres muscle depending on curveball pitching.

In this study, the variation in the results of flexor carpi ulnaris and extensor carpi ulnaris in curveball pitching was larger than that in fastball pitching. This result may be attributed to the fact that there are individual differences in the way the curveball is gripped. Previous studies reported the same individual differences in ball gripping. Additionally, variations in ball rotation characteristics have been observed [32,33]. Therefore, it is thought that different ball gripping styles lead to different finger and wrist joint kinematics during the pitching motion. The present study suggests that curveball pitching may involve significant differences in forearm muscle activities from player to player and that the risk of medial elbow injuries caused by the overuse of the forearm muscles may also differ from fastball pitching.

The results of this study showed that curveball pitching causes an increase in fore-arm-specific muscle activity, namely that of the pronator teres muscle, which may increase the possibility that repetitive curveball pitching may contribute to the risk of throwing in-juries, including medial elbow joint disorders and pronator teres syndrome. This indicates the need to actively investigate whether limiting the amount that the breaking ball pitching throw is used has a preventive effect, while teaching correct gripping techniques to prevent elbow joint injuries on coaching and in rehabilitation. In particular, translating and communicating this information to athletic trainers, coaches, physical therapists, or other staff members involved in providing instruction to athletes may contribute to reducing the incidence of throwing injuries in athletes, extending athlete longevity, and improving throwing performance.

This study contains two main limitations. Firstly, we were unable to investigate the rotational characteristics of the ball. Evaluating ball rotational characteristics would allow for a more detailed examination of forearm muscle activation using different gripping techniques. Secondly, the sample size was relatively small as this was a pilot study. Therefore, careful consideration is needed when interpreting the significance of the results of the present study, and statistically stronger results should be presented in future studies with large-scale designs. Furthermore, future studies should aim to investigate pronator teres muscle activation during breaking ball pitching and assess the risk of throwing injury in baseball pitchers on a larger scale.

## 5. Conclusions

The findings of this study suggest that curveball pitching is a factor in causing the over-activation of the pronator teres muscle. Increased muscle activity in the pronator teres during the curveball pitch may contribute to stiffness and induce pronator teres syndrome or medial elbow injuries related to the overuse of the pronator teres. Therefore, controlling curveball throwing movements contributes to player coaching and conditioning focusing on the prevention of elbow joint disorders and pronator teres syndrome. Future studies should aim to investigate pronator teres muscle activation during breaking ball pitching and assess the risk of throwing injury in baseball pitchers on a larger scale.

## Figures and Tables

**Table 1:** The average in EMG values (%MVC) during fastball and curveball pitching.

	Fastball	Curveball	Mean Difference (95% CI ^h^)	*t* Value	*p* Value	Effect SizeCohen”s *d*
	Mean ± SD ^g^	Mean ± SD ^g^				
BRD ^a^	234.8 ± 125.8	220.8 ± 74.5	14.0 (−67.7–95.6)	0.39	0.71	0.12
PT ^b^	165.4 ± 114.4	192.7 ± 91.9	−27.4 (−71.5–16.8)	−1.40	0.20	−0.42
FCR ^c^	114.9 ± 76.6	148.1 ± 42.5	33.2 (−79.6–13.2)	−1.62	0.14	−0.49
FCU ^d^	114.3 ± 82.9	181.0 ± 134.7	−66.7 (−147.0–13.6)	−1.88	0.09	−0.57
ECU ^e^	135.7 ± 127.1	155.9 ± 144.0	−20.2 (−169.0–128.6)	−0.31	0.77	−0.09
ECR ^f^	104.9 ± 90.5	111.9 ± 80.1	−7.0 (−102.0–87.9)	−0.17	0.87	−0.05

^a^ BRD: brachioradialis muscle; ^b^ PT: pronator teres muscle; ^c^ FCR: flexor carpi radialis; ^d^ FCU: flexor carpi ulnaris; ^e^ ECU: extensor carpi ulnaris; ^f^ ECR: extensor carpi radialis; ^g^ SD: standard deviation; ^h^ CI: confidence interval.

**Table 2:** The peak in EMG values (%MVC) during fastball and curveball pitching.

	Fastball	Curveball	Mean Difference (95% CI ^h^)	*t* Value	*p* Value	Effect Size Cohen”s *d*
	Mean ± SD ^g^	Mean ± SD ^g^				
BRD ^a^	658.4 ± 329.3	598.1 ± 218.3	60.4 (−187.2–307.9)	0.55	0.60	0.17
PT ^b^	340.1 ± 226.2	450.4 ± 193.7	−110.3 (−209.6–−10.9)	−2.51	0.03 *	−0.79
FCR ^c^	260.2 ± 147.3	346.0 ± 175.8	−85.8 (−219.2–47.5)	−1.46	0.18	−0.46
FCU ^d^	268.6 ± 260.5	428.6 ± 429.2	−160.1 (−422.7–102.6)	−1.38	0.20	−0.47
ECU ^e^	308.6 ± 438.8	380.8 ± 371.8	−72.2 (−531.1–386.7)	−0.36	0.73	−0.11
ECR ^f^	233.2 ± 229.1	269.3 ± 233.1	−36.1 (−297.9–225.7)	−0.31	0.76	0.10

^a^ BRD: brachioradialis muscle; ^b^ PT: pronator teres muscle; ^c^ FCR: flexor carpi radialis; ^d^ FCU: flexor carpi ulnaris; ^e^ ECU: extensor carpi ulnaris; ^f^ ECR: extensor carpi radialis; ^g^ SD; standard deviation; ^h^ CI; confidence interval; *: statistical significance (*p* < 0.05).

## Data Availability

The data presented in this study are available upon request from the corresponding author. The data were not publicly available because of privacy concerns.
